# Medication adherence and treatment persistence following switching from vitamin K antagonists to direct oral anticoagulants: a systematic review

**DOI:** 10.1186/s12872-026-06398-0

**Published:** 2026-08-01

**Authors:** Ahmed Alaqab, Mohammed Alfaqeeh, Rosnani Hashim, Maali Mustafa, Anas Hamad, Niveen M. E. Abu-Rmeileh, Rizky Abdulah, Sofa D. Alfian

**Affiliations:** 1 Department of Pharmacy, Faculty of Medical Sciences, Al-Ruwad University, Taiz, Republic of Yemen; 2 Department of Pharmacology and Clinical Pharmacy, Faculty of Pharmacy, Universitas Padjadjaran, Bandung, Indonesia; 3 Department of Hospital and Clinical Pharmacy, Faculty of Pharmacy, University of Cyberjaya, Cyberjaya, Malaysia; 4 Drug Supply Department, Hamad Medical Corporation, Doha, Qatar; 5 College of Pharmacy, QU Health, Qatar University, Doha, Qatar; 6 Department of Public Health, College of Health Sciences, QU Health, Qatar University, Doha, Qatar; 7 Centre of Excellence for Pharmaceutical Care Innovation, Universitas Padjadjaran, Bandung, Indonesia; 8 Center for Health Technology Assessment, Universitas Padjadjaran, Bandung, Indonesia

**Keywords:** Direct oral anticoagulants, Vitamin K antagonists, Adherence, Persistence, Anticoagulation, Atrial fibrillation

## Abstract

**Background:**

Switching from vitamin K antagonists (VKAs) to direct oral anticoagulants (DOACs) has become increasingly common because of the limitations associated with VKA therapy. However, adherence and persistence after switching remain important determinants of treatment effectiveness. This systematic review evaluated adherence and persistence outcomes following switching from VKAs to DOACs and summarized the reported reasons for switching.

**Methods:**

A systematic review was conducted in accordance with PRISMA guidelines. PubMed and Scopus were searched for studies published between January 2016 and April 2026 evaluating adherence or persistence following switching from VKAs to DOACs. Eligible studies included randomized and observational studies involving adult patients with atrial fibrillation, venous thromboembolism, or other indications requiring anticoagulation therapy. Data on study characteristics, adherence and persistence outcomes (including proportion of days covered, medication possession ratio, and discontinuation measures), and reasons for switching were extracted and synthesized narratively.

**Results:**

Sixteen studies were included. Adherence was assessed using both objective refill-based measures and subjective patient-reported tools. Objective adherence rates were generally moderate to high, with the proportion of patients achieving proportion of days covered (PDC) ≥ 80% ranging from 76% to 97.5% across most studies. Studies using subjective patient-reported instruments, including MMAS-8, ACTS, and PACT-Q2, reported more variable adherence levels. Persistence rates ranged from 54% to 94%, although substantial variability in persistence definitions and follow-up duration limited direct comparison across studies. Poor international normalized ratio control and low time in therapeutic range were the most commonly reported reasons for switching from VKAs to DOACs.

**Conclusions:**

Studies evaluating switching from VKAs to DOACs generally reported moderate-to-high objective adherence rates, although persistence outcomes and subjective adherence measures were more variable. Methodological heterogeneity across studies highlights the need for standardized evaluation approaches and continued adherence-support strategies after switching.

**Supplementary Information:**

The online version contains supplementary material available at https://doi.org/10.1186/s12872-026-06398-0.

## Introduction

Atrial fibrillation (AF) and venous thromboembolism (VTE), including deep vein thrombosis and pulmonary embolism, are among the most common cardiovascular conditions requiring short- or long-term oral anticoagulation therapy [1, 2]. In these populations, anticoagulant treatment plays a central role in reducing the risk of ischemic stroke, systemic embolism, recurrent thromboembolism, and related mortality [3]. For decades, vitamin K antagonists (VKAs), particularly warfarin, were the standard oral anticoagulants used in clinical practice [4]. However, their use is complicated by a narrow therapeutic range, substantial interindividual dose variability, frequent international normalized ratio (INR) monitoring, numerous food and drug interactions, and the need for ongoing dose adjustment [5, 6]. These limitations may impose a significant treatment burden on both patients and healthcare systems [7].

Direct oral anticoagulants (DOACs), including apixaban, rivaroxaban, dabigatran, and edoxaban, have substantially changed the management of thromboembolic disorders [8]. Compared with VKAs, DOACs offer fixed-dose regimens, fewer clinically relevant interactions, rapid onset and offset of action, and no requirement for routine coagulation monitoring [9]. Large randomized trials and subsequent real-world studies have shown that DOACs are at least non-inferior to VKAs for thromboembolic prevention, with favorable safety outcomes in many patient groups [10]. As a result, switching from VKA therapy to a DOAC has become increasingly common, particularly in patients with unstable INR control, poor time in therapeutic range (TTR), inconvenience related to monitoring, adverse effects, or patient preference [11].

Despite these practical advantages, the effectiveness of DOAC therapy remains highly dependent on medication adherence and treatment persistence [12]. According to the ABC taxonomy proposed by Vrijens et al., adherence refers to the process by which patients take medications as prescribed, particularly the extent to which actual dosing corresponds to the prescribed regimen (implementation), whereas persistence refers to the duration of time from treatment initiation to discontinuation [13]. Because DOACs have relatively short half-lives and predictable pharmacokinetic profiles, missed doses or premature discontinuation may rapidly reduce anticoagulant protection [14]. In contrast to warfarin therapy, where suboptimal control may be detected through INR monitoring, non-adherence to DOAC therapy may remain clinically silent until thromboembolic or bleeding complications occur [15]. Therefore, maintaining consistent long-term medication use is essential to achieve the expected benefits of therapy after switching [16].

Importantly, switching from a VKA to a DOAC does not necessarily resolve the underlying barriers associated with long-term medication use. Patients who experienced poor anticoagulation control while receiving VKAs may continue to face challenges related to health literacy, treatment beliefs, polypharmacy, cognitive impairment, cost, regimen complexity, or limited healthcare access [17, 18]. In addition, some DOAC regimens require twice-daily dosing, which may influence medication implementation differently from once-daily alternatives [19]. Furthermore, previous experiences with anticoagulation monitoring, dose adjustments, treatment dissatisfaction, or unstable INR control may continue to influence medication-taking behavior after switching [20]. Some patients may experience improved treatment satisfaction and convenience after transition to DOACs, whereas others may carry forward pre-existing adherence-related behavioral patterns or concerns regarding anticoagulation therapy [21]. Consequently, patients who switch therapy represent a clinically and behaviorally distinct population whose post-switch adherence and persistence patterns may differ from those of anticoagulant-naïve DOAC users.

Previous studies have evaluated adherence and persistence among general users of DOAC therapy [22–24]. However, evidence specifically focused on patients transitioning from VKAs to DOACs remains limited, and the extent to which switching improves adherence and persistence has not been comprehensively synthesized in a structured systematic review [25]. A clearer understanding of treatment behavior in this population is clinically relevant for optimizing patient selection for switching from VKAs to DOACs, counseling, follow-up strategies, and long-term anticoagulation outcomes [26]. Therefore, this systematic review aimed to evaluate medication adherence and treatment persistence following switching from VKAs to DOACs, to summarize the reasons for switching, and to provide a comprehensive synthesis of available evidence across AF and VTE populations.

## Methods

### Study design and registration

This study was designed as a systematic review to evaluate medication adherence and treatment persistence following switching from VKAs to DOACs. The review was conducted in accordance with the Preferred Reporting Items for Systematic Reviews and Meta-Analyses (PRISMA) statement [27]. The review protocol was prospectively registered in the International Prospective Register of Systematic Reviews International Prospective Register of Systematic Review (PROSPERO) (CRD420261381371) [28]. A completed PRISMA checklist is also provided in Supplementary File 1.

### Search strategy

A systematic literature search was conducted in PubMed and Scopus to identify studies evaluating medication adherence and treatment persistence following switching from VKAs to DOACs. These databases were selected because of their broad biomedical and multidisciplinary coverage, and index the majority of peer-reviewed literature relevant to anticoagulation therapy, medication adherence, and cardiovascular research. Searches covered studies published from January 2016 to April 2026. The search strategy combined controlled vocabulary terms, where applicable, and free-text keywords related to VKAs, DOACs, medication-taking behavior, and treatment switching. Boolean operators (“AND” and “OR”) were applied according to the requirements of each database to optimize search sensitivity and specificity. Only studies published in English were included, which may have led to the exclusion of relevant studies published in other languages. The full electronic search strategies for both databases are provided in Supplementary File 2 (Table S1).

### Eligibility criteria and study selection

Study eligibility was predefined using the PICOS framework. Eligible studies included adult populations with AF, DVT, pulmonary embolism (PE), VTE, or other indications requiring oral anticoagulation who had been switched or transitioned from VKA therapy to any DOAC. Switching was defined as the transition from any VKA (e.g., warfarin, acenocoumarol, or phenprocoumon) to any approved DOAC, irrespective of the specific anticoagulant agent used. No minimum duration of VKA therapy prior to switching was specified because this information was inconsistently reported across the primary studies. Therefore, studies were considered eligible if they evaluated post-switch adherence and/or persistence regardless of the duration of prior VKA exposure. As medication adherence and treatment persistence are behavioral outcomes applicable across different indications for oral anticoagulation, studies involving these approved clinical indications were considered eligible. The underlying anticoagulation indication was extracted for each included study; however, most primary studies reported adherence and persistence outcomes for the overall study population rather than separately for AF and VTE, limiting indication-specific synthesis. When reported, the reasons for switching (e.g., poor INR control, low time in therapeutic range, bleeding concerns, treatment burden, physician recommendation, or patient preference) were extracted and summarized narratively. However, because these reasons were inconsistently reported and defined across studies, they were not incorporated into the study eligibility criteria or subgroup analyses.

When available, comparator groups included continued VKA therapy, de novo DOAC initiation, alternative DOAC agents, or low-molecular-weight heparin (LMWH). However, studies without a comparator group were also eligible if they reported post-switch adherence and/or persistence outcomes. Accordingly, results were narratively synthesized for the switched population rather than compared across treatment groups. Because this review synthesized published study-level evidence rather than individual patient data, study-specific eligibility criteria regarding patients with mechanical heart valves, moderate-to-severe mitral stenosis, or other contraindications to anticoagulation were retained as reported in the original studies, and no additional patient-level exclusion criteria were imposed.

Primary outcomes were medication adherence and treatment persistence. Adherence outcomes included measures of implementation of the prescribed dosing regimen, such as proportion of days covered (PDC), medication possession ratio (MPR), refill adherence, and other validated adherence measures. Persistence outcomes included discontinuation rates, time to discontinuation, duration of therapy, or proportion of patients remaining on treatment over time. Secondary outcomes included patient-reported adherence, treatment satisfaction, healthcare utilization, and clinically relevant outcomes associated with non-adherence when reported.

Randomized controlled trials (RCTs), prospective and retrospective cohort studies, registry analyses, cross-sectional studies reporting adherence or persistence outcomes, and other observational studies using real-world or clinical trial data were eligible. Both RCTs and observational studies were included to capture evidence from controlled clinical settings as well as real-world medication-taking behavior and long-term treatment patterns following switching from VKAs to DOACs. Reviews, editorials, conference abstracts without sufficient data, case reports, case series, protocol-only publications, and studies lacking extractable outcomes were excluded.

All retrieved records were imported into Rayyan, and duplicate records were removed prior to screening. Study selection was conducted in two phases: (1) title and abstract screening and (2) full-text eligibility assessment. Screening was performed independently by two reviewers (AA and MA), and disagreements were resolved through discussion with involvement of a third reviewer (SDA) when required.

### Data extraction

Data extraction was conducted independently by two reviewers using a standardized, piloted data collection form developed in Microsoft Excel. Any discrepancies were resolved through discussion, with consultation from a third reviewer when necessary. Extracted data included study characteristics (first author and year, country, and study design), population and clinical indication, sample size, intervention characteristics (switching from VKA to DOAC), follow-up duration, adherence measurement methods (e.g., PDC, MPR, refill adherence), persistence measurement methods and definitions (e.g., discontinuation rates, time to discontinuation, persistence duration), and reported adherence or persistence outcomes where applicable.

Outcome data extracted from eligible studies included medication adherence and treatment persistence measures such as PDC, MPR, refill adherence, discontinuation rates, persistence rates at predefined time points, time to discontinuation, and other validated adherence indicators where applicable. Additional outcomes, patient-reported adherence using validated instruments, such as the Anti-Clot Treatment Scale (ACTS), the Morisky Medication Adherence Scale (MMAS-8), and the Perception of Anticoagulant Treatment Questionnaire (PACT-Q2), treatment satisfaction, and factors influencing adherence or persistence (e.g., clinical, behavioral, or healthcare-related factors), were also extracted when reported. Where studies reported multiple adherence measures, definitions, variables associated with medication adherence or persistence or time points, all relevant data were extracted and reported descriptively.

### Risk of bias assessment

Methodological quality of the included studies was assessed independently by two (AA and MA) using validated, design-specific instruments according to study methodology. Any discrepancies in quality assessment were resolved through discussion until consensus was reached, with consultation from a third reviewer (SDA) when necessary. Randomized controlled trials (RCTs) were evaluated using the Cochrane Risk of Bias Tool version 2 (RoB 2), which assesses bias arising from the randomization process, deviations from intended interventions, missing outcome data, outcome measurement, and selective reporting [27]. Given the nature of adherence research, findings from RCTs were interpreted with consideration of potential trial-related behavioral effects, including closer patient monitoring and follow-up, which may result in higher adherence estimates compared with real-world observational studies. Observational cohort studies, quasi-experimental studies, and registry-based studies were assessed using the Newcastle–Ottawa Scale (NOS), which evaluates methodological quality across three domains: selection, comparability, and outcome [29]. The NOS scores were interpreted as follows: scores of 7–9 indicated low risk of bias, 5–6 indicated moderate risk of bias, and scores below 5 indicated high risk of bias. Cross-sectional studies were assessed using the Appraisal tool for Cross-Sectional Studies (AXIS) [30]. As the AXIS instrument does not generate an overall numerical score, methodological quality was evaluated descriptively across its domains, with emphasis on study design, sampling strategy, measurement validity, and potential sources of bias.

Each study was judged according to the criteria of the relevant tool, leading to domain-level and overall risk-of-bias judgments where applicable. Any disagreements between reviewers were resolved through discussion, with consultation from a third reviewer when necessary. Certainty of evidence using the GRADE approach was not assessed because substantial methodological heterogeneity across study designs, adherence definitions, and outcome measures precluded quantitative synthesis.

### Data synthesis and statistical analysis

A narrative synthesis was undertaken for all included studies and structured according to study design, population characteristics, adherence measures, and persistence outcomes. Study characteristics, adherence definitions, follow-up duration, and reported findings were summarized descriptively. Extracted data were managed using Microsoft Excel. Risk-of-bias assessments were conducted using the relevant appraisal tools, and graphical visualizations of risk-of-bias judgments were generated using appropriate software packages.

Due to substantial heterogeneity in study designs, adherence definitions, persistence measures, and reporting approaches, quantitative synthesis (meta-analysis) was not performed. Instead, adherence and persistence outcomes were summarized descriptively across studies. Adherence results were reported as mean PDC/MPR values or proportions of patients achieving predefined thresholds (e.g., PDC ≥ 80%), while persistence outcomes were summarized according to study-specific definitions and follow-up durations. To facilitate interpretation of the findings, methodological heterogeneity was explored qualitatively by comparing adherence and persistence definitions, measurement methods (objective versus subjective), follow-up duration, study population, and reported outcome ranges across the included studies.

## Results

### Study selection

**Fig. 1:**
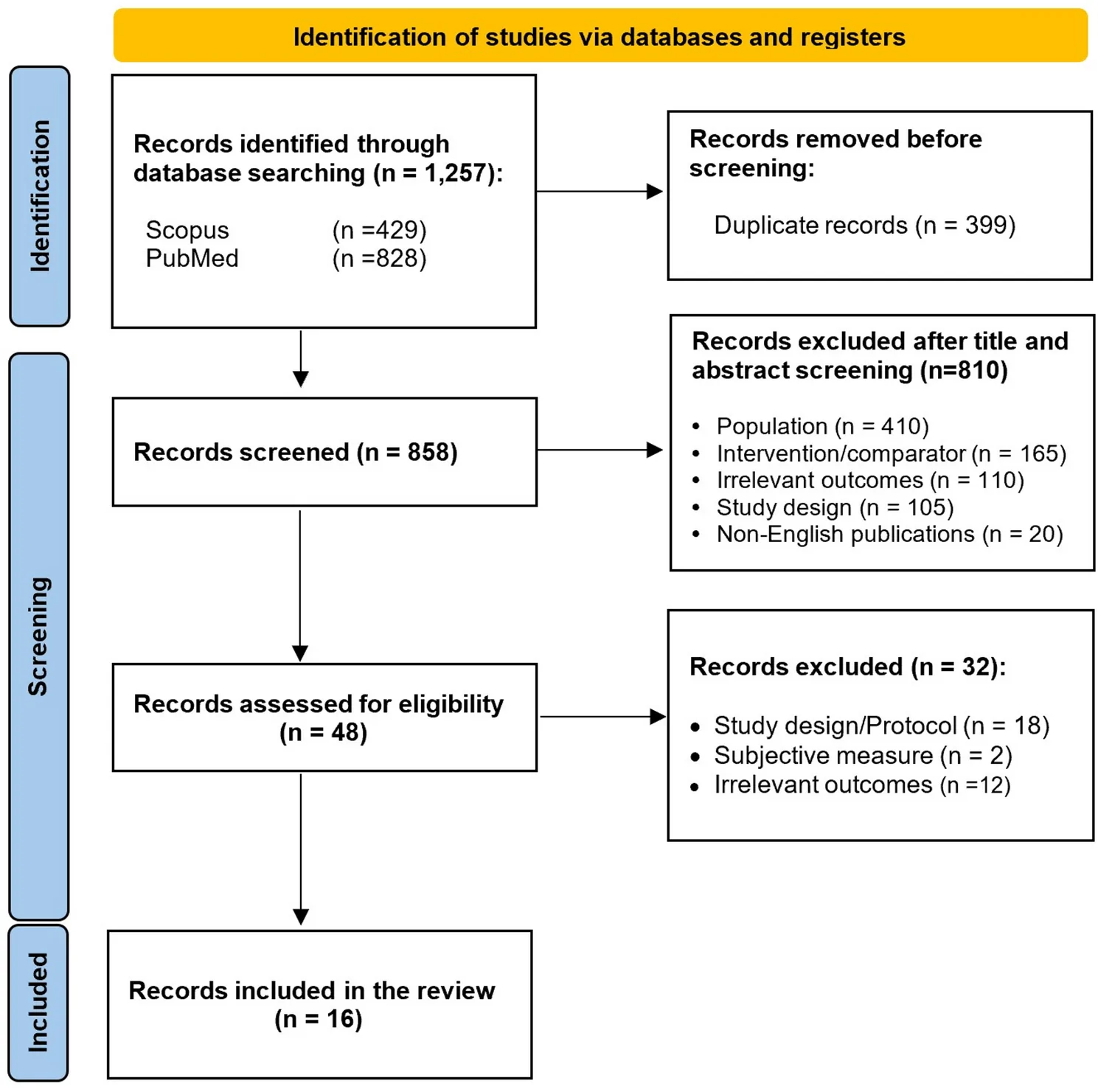
PRISMA 2020 flow diagram of the study selection process

The database search identified a total of 1,257 records, including 429 from Scopus and 828 from PubMed. After removal of 399 duplicate records, 858 unique records remained for title and abstract screening. Of these, 810 records were excluded as clearly irrelevant to the review question. A total of 48 full-text articles were assessed for eligibility. Following full-text review, 32 studies were excluded, primarily due to irrelevant study design or protocol-only publications (*n* = 18), or outcomes not relevant to the review objectives (*n* = 14). Ultimately, 16 studies met the predefined eligibility criteria and were included in the systematic review. The study selection process is summarized in Fig. 1.

### Characteristics of included studies

A summary of the characteristics of the included studies is presented in Table 1. The 16 included studies were conducted across multiple geographic regions, with the majority originating from Europe, including studies from the Netherlands [31–34], France [35–37], Italy [38], and the United Kingdom [39]. A substantial proportion of studies were also conducted in North America, particularly in the United States [40–42], and Canada [43]. Additional studies were conducted in Asia, including Japan [44] and Taiwan [45], as well as one international study [46]. In terms of study design, retrospective cohort studies were the most common, accounting for 5 of 16 studies [32, 33, 40, 42, 43]. Three studies were prospective cohort studies [35, 39, 44], two were prospective observational studies [38, 45], and one was a prospective registry-based cohort study [46]. Cross-sectional studies also accounted for 3 studies [32, 36, 37, 45]. The remaining studies included one RCT [34], and one quasi-experimental pre–post observational study [41].

**Table 1:** Characteristics of the included studies

Study (Author, Year)	Country	Study Design	Population	Sample Size (Switchers)	Switching Pattern	Assessment Period / Follow-up (months)	DOAC Agent
Toorop et al., 2022 [32]	Netherlands	Retrospective cohort study	AF	3696	VKA → DOAC	48	Mixed DOACs
Pundi et al., 2021 [40]	USA	Retrospective cohort study	AF	32,377	Warfarin → DOAC	12	Mixed DOACs
Toorop et al., 2020 [31]	Netherlands	Cross-sectional study	AF	1399	VKA → DOAC	16*	Mixed DOACs
Hanon et al., 2016 [35]	France	Prospective cohort study	AF	405	VKA → Rivaroxaban	6	Rivaroxaban
Inoue et al., 2016 [44]	Japan	Prospective cohort study	AF	1701	Warfarin → Dabigatran	24	Dabigatran
Elling et al., 2024 [33]	Netherlands	Retrospective cohort study	AF/VTE	437	VKA → DOAC	34	Mixed DOACs
Douros et al., 2017 [43]	Canada	Retrospective cohort study	AF	4763	VKA → DOAC	36	Mixed DOACs
Bartoli-Abdou et al., 2021 [39]	UK	Prospective cohort study	AF/VTE	220	VKA → DOAC	12	Mixed DOACs
Pham Nguyen et al., 2020 [41]	USA	Quasi-experimental cohort study	AF	691	Warfarin → DOAC	12	Mixed DOACs
Lee et al., 2021 [45]	Taiwan	Cross-sectional study	AF	139	VKA → Dabigatran	6*	Dabigatran
Miert et al., 2019 [34]	Netherlands	Randomized controlled trial	AF	121	VKA → DOAC	12	Apixaban
Yang et al., 2020 [46]	International	Prospective registry-based cohort study	Mixed	150	VKA → DOAC	12	Mixed DOACs
Mews et al., 2021 [42]	USA	Retrospective cohort study	AF	66	Warfarin → DOAC	12	Mixed DOACs
Benzimra et al., 2018 [36]	France	Cross-sectional study	AF/VTE	50	VKA → DOAC	48*	Mixed DOACs
Serrao et al., 2020 [38]	Italy	Prospective cohort study	AF/VTE	107	VKA → DOAC	39	Mixed DOACs
Keita et al., 2017 [37]	France	Cross-sectional study	VTE	10	VKA → DOAC	3*	Mixed DOACs

*Abbreviations*: *AF *atrial fibrillation, *VTE *venous thromboembolism, *VKA *vitamin K antagonist, *DOAC *direct oral anticoagulant, *USA *United States of America, *UK *United Kingdom

* indicates assessment period for cross-sectional studies

The study populations were predominantly composed of patients with AF, which was the sole indication in 11 studies (68.8%). Mixed populations of AF and VTE were included in 4 studies (25%) [33, 36, 38, 39], while one study (6.3%) focused exclusively on VTE patients [37]. Sample sizes varied substantially across studies, ranging from 10 [37] to 32,377 patients [40]. Most studies evaluated patients who were switched from VKAs to DOACs, with several studies specifically examining transitions from warfarin to individual DOAC agents such as dabigatran or rivaroxaban [35, 44, 45]. Follow-up duration after switching varied considerably, ranging from 3 [37] to 48 months [36]. Regarding anticoagulant exposure, the majority of studies (75%) evaluated mixed DOAC populations, including combinations of apixaban, rivaroxaban, dabigatran, and edoxaban. A smaller number of studies focused on single DOAC agents, including rivaroxaban [35], dabigatran [44, 45], and apixaban [34].

### Reasons for switching to DOACs

Among the studies that reported reasons for switching (*n* = 12), the most commonly reported reason for switching from VKAs to DOACs was poor anticoagulation control, typically reflected by low time in therapeutic range (TTR) or unstable INR values [32, 33, 35, 38–40]. Physician-driven decisions were also frequently reported, including clinical judgment and treatment preference [31, 46]. In addition, patient-related factors such as convenience and the desire to avoid routine monitoring were identified as contributing reasons for switching [36, 37]. Less commonly, switching was triggered by clinical events or healthcare-related factors, such as hospitalization or efforts to reduce clinic visits [41, 42]. Overall, these findings suggest that switching to DOACs is primarily driven by suboptimal anticoagulation control and the perceived advantages of DOACs in terms of convenience and reduced monitoring burden.

### Adherence outcomes

Adherence measures were reported across the included studies using heterogeneous assessment approaches, with quantitative results summarized in Table 2.

**Table 2:** Adherence outcomes across included studies

Study	Measure Type	Measure Definition	Mean PDC/MPR (%)	PDC ≥ 80% (%)
Benzimra et al., 2018 [36]	Subjective	MMAS-8 / PACT-Q2	NR	68%
Toorop et al., 2020 [31]	Subjective	Self-reported adherence questionnaire	NR	86%
Hanon et al., 2016 [35]	Subjective	ACTS / interview	NR	97.5%
Inoue et al., 2016 [44]	Subjective	Clinician-reported adherence	NR	92%
Lee et al., 2021 [45]	Subjective	PACT-Q2	NR	NR
Keita et al., 2017 [37]	Subjective	MMAS-8	NR	50%
Pundi et al., 2021 [40]	Objective	PDC ≥ 80%	86%	76%
Elling et al., 2024 [33]	Objective	PDC ≥ 90%	NR	80%
Pham Nguyen et al., 2020 [41]	Objective	PDC ≥ 80%	81.7%	NR
Miert et al., 2019 [34]	Objective	PDC ≥ 80%	NR	94%
Mews et al., 2021 [42]	Objective	Refill-based adherence (EHR)	NR	NR
Yang et al., 2020 [46]	Mixed	Pharmacy + MMAS-8	NR	93%
Bartoli-Abdou et al., 2021 [39]	Mixed	PDC + MMAS-8	92%	77%
Serrao et al., 2020 [38]	Mixed	Survey + plasma	NR	90%

*Abbreviations*: *PDC *proportion of days covered, *MPR *medication possession ratio, *MMAS-8 *8-item Morisky Medication Adherence Scale, *ACTS *Anti-Clot Treatment Scale, *PACT-Q2 *Perception of Anticoagulant Treatment Questionnaire, *EHR *electronic health record, *NR *not reported, *RCT *randomized controlled trial

Across studies, adherence was assessed using both objective measures, such as PDC and MPR, and subjective approaches, including validated patient-reported questionnaires. Objective adherence measures were most commonly derived from administrative or pharmacy claims databases [33, 34, 40–43], whereas subjective measures included validated tools such as the Anti-Clot Treatment Scale (ACTS), the Morisky Medication Adherence Scale (MMAS-8), and the Perception of Anticoagulant Treatment Questionnaire (PACT-Q2) [35–37, 45]. Several studies combined both approaches, reflecting attempts to capture both behavioral and clinical dimensions of adherence [38, 39, 46].

Quantitative adherence outcomes varied according to the measurement approach used (Table 2). Among studies using subjective adherence measures, adherence assessed by validated questionnaires or interviews ranged from 50% to 97.5%, with the highest adherence reported by Hanon et al. (97.5%) [35], followed by Inoue et al. (92%) [44] and Toorop et al. (86%) [31], whereas lower adherence estimates were observed in Benzimra et al. (68%) [36] and Keita et al. (50%) [37].

Among studies using objective adherence measures, mean PDC/MPR values ranged from 81.7% [41] to 86% [40]. Studies reporting adherence using predefined PDC thresholds demonstrated generally favorable adherence, ranging from 76% [40] to 94% [34], with Miert et al. reporting the highest adherence (94%) [34], followed by Elling et al. (80%) [33].

Studies employing mixed adherence measures also demonstrated generally favorable adherence. Bartoli-Abdou et al. reported a mean adherence of 92% with 77% of patients achieving PDC ≥ 80% [39], while Yang et al. and et al. reported adherence rates of 93% and 90%, respectively [38, 46].

### Persistence outcomes

Persistence outcomes were reported in a subset of included studies (*n* = 7), as summarized in Table 3. Follow-up duration ranged from 6 to 48 months, and persistence was assessed using heterogeneous definitions, including prescription-gap methods, discontinuation-based measures, Kaplan–Meier estimates, self-reported persistence, and electronic health record–based assessments. No consistent relationship between follow-up duration and persistence could be identified because persistence was defined using different methodologies across studies, precluding meaningful comparison. Most studies reported persistence outcomes for the overall study population, preventing separate synthesis according to anticoagulation indication.

**Table 3:** Persistence following switching to Direct Oral Anticoagulants (DOACs)

Study	Persistence Definition	Follow-up Duration	Persistence Rate (%)
Hanon et al., 2016 [35]	Kaplan–Meier estimated continuation	6 months	88.7%
Lee et al., 2021 [45]	100% minus discontinuation	6 months	78.5%
Mews et al., 2021 [42]	Active EHR medication list	12 months	89.4%
Toorop et al., 2020 [31]	Self-reported persistence	16 months	94%
Elling et al., 2024 [33]	180-day prescription gap	34 months	82.2%
Douros et al., 2017 [43]	30-day grace period	36 months	54%
Toorop et al., 2022 [32]	100-day prescription gap	48 months	89.4%

*Abbreviations*: *EHR *electronic health record

Persistence definitions are reported as described in the original studies

Persistence estimates ranged from 54% to 94%. The highest persistence was observed in Toorop et al. (94%) [31], followed by Toorop et al. and Mews et al., both reporting persistence rates of 89.4% [32, 42]. Similarly, high persistence was reported in Hanon et al. (88.7%) [35]. Moderate persistence rates were observed in Elling et al. (82.2%) [33], and Lee et al. (78.5%) [45], while substantially lower persistence was reported in Douros et al. (54%) [43]. Importantly, persistence definitions varied considerably across studies, including prescription gap thresholds (e.g., 100-day or 180-day gaps), discontinuation-based measures, Kaplan–Meier survival estimates, and electronic health record–based definitions. This heterogeneity limits direct comparability across studies and should be considered when interpreting the findings.

### Factors associated with adherence and persistence

Several studies evaluated factors associated with medication adherence and/or persistence following switching from VKAs to DOACs, although the variables assessed differed considerably across studies. Reported factors included patient characteristics (age, thromboembolic risk), treatment-related factors (dosing frequency, pre-switch anticoagulation control, medication beliefs, bleeding-related hospitalization), healthcare-related factors (specialist follow-up and pharmacist-led management), and socioeconomic factors (income deprivation and medication cost). However, these determinants were evaluated inconsistently, and several studies reported only descriptive adherence or persistence outcomes without formal predictor analyses. Consequently, no quantitative synthesis of associated factors was performed. A detailed summary is presented in Supplementary File 2 (Table S2).

### Quality assessment

A total of 11 studies were assessed using the NOS. Overall, the methodological quality ranged from moderate to high. Five studies (45.5%) were judged to be at low risk of bias [32, 33, 40, 41, 43], demonstrating strong selection methods, appropriate cohort definitions, and relatively robust outcome assessment. Five studies (45.5%) were classified as having moderate risk of bias [35, 39, 42, 44, 46], primarily due to limited adjustment for confounding variables and potential selection bias. One study was judged to be at high risk of bias, mainly due to small sample size, lack of comparability, and limited methodological rigor [38]. Detailed NOS assessments for individual studies are presented in Table 4.

**Table 4:** Risk of bias assessment of cohort and quasi-experimental studies using the Newcastle–Ottawa Scale (NOS)

Study	Selection (0–4)	Comparability (0–2)	Outcome (0–3)	Total (0–9)	Overall Risk	Key Justification
Toorop et al., 2022 [32]	4	2	2	8	Low	Large registry, good selection, objective outcomes
Pundi et al., 2021 [40]	4	2	3	9	Low	Large database, strong adjustment, robust outcome
Hanon et al., 2016 [35]	3	1	2	6	Moderate	Confounding likely, limited adjustment
Inoue et al., 2016 [44]	3	0	2	5	Moderate	No comparability adjustment
Elling et al., 2024 [33]	4	1	2	7	Low	Good design, partial confounding control
Douros et al., 2017 [43]	4	1	2	7	Low	Large dataset, some residual confounding
Bartoli-Abdou et al., 2021 [39]	3	1	2	6	Moderate	Limited comparability
Pham Nguyen et al., 2020 [41]	4	2	2	8	Low	Quasi-experimental with strong structure
Yang et al., 2020 [46]	3	0	2	5	Moderate	Registry without adjustment
Mews et al., 2021 [42]	3	0	2	5	Moderate	Limited control of confounders
Serrao et al., 2020 [38]	2	0	1	3	High	Small sample, weak methodology

Four studies were assessed using the AXIS tool [31, 36, 37, 45]. Overall, these studies demonstrated moderate methodological quality. All studies clearly defined their objectives and used validated instruments to assess adherence and treatment satisfaction. However, some limitations were identified. All studies relied on self-reported measures, introducing potential reporting bias. Additionally, non-response bias was not adequately addressed across all studies, and sampling methods were often not fully representative of the target population. Small sample sizes and single-center designs were also noted in some studies, limiting generalizability [36, 37]. Detailed AXIS assessments are provided in Supplementary File 2 (Table S3).

**Fig. 2:**
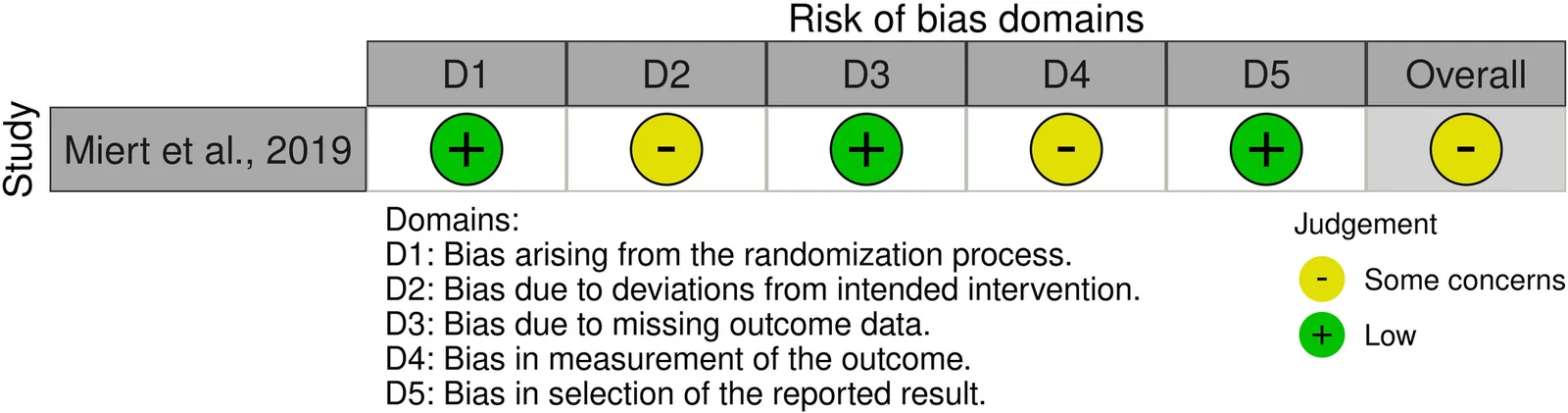
Risk of bias assessment of the randomized controlled trial using the Cochrane Risk of Bias Tool version 2 (RoB 2). Domain-level and overall risk-of-bias judgments for the included randomized controlled trial [34]. Domains assessed include bias arising from the randomization process (D1), deviations from intended interventions (D2), missing outcome data (D3), measurement of the outcome (D4), and selection of the reported result (D5). Green (+) indicates low risk of bias, while yellow (−) indicates some concerns

One RCT was assessed using the RoB 2 tool and was judged to have some concerns overall [34]. While the study demonstrated low risk of bias in domains related to randomization and reporting, concerns were identified in the domains of deviations from intended interventions and outcome measurement, likely due to lack of blinding and the nature of adherence assessment. A graphical summary of the RoB 2 assessment is presented in Fig. 2. Overall, the risk-of-bias assessments indicated that the included evidence was of predominantly moderate methodological quality. These findings were considered when interpreting the overall results of this review.

## Discussion

This systematic review evaluated adherence and persistence outcomes among patients switching from VKAs to DOACs across 16 studies involving diverse clinical settings and study designs. Overall, adherence and persistence rates following switching were generally reported as moderate to high, although substantial variability was observed according to the assessment method, follow-up duration, and patient population. Studies using objective refill- or claims-based measures generally reported higher adherence estimates compared with self-reported assessments. Interpretation of these findings requires caution, as most included studies were observational in design and lacked direct pre- and post-switch adherence comparisons. Poor INR control and low TTR were the most frequently reported reasons for switching from VKAs to DOACs. However, despite the simplified management associated with DOAC therapy, variability in persistence rates and adherence thresholds suggests that switching alone may not fully overcome barriers to long-term anticoagulant adherence.

The reasons underlying switching from VKAs to DOACs provide important context for interpreting both adherence and persistence outcomes observed in the included studies. Across the reviewed literature, poor INR control and low TTR were the most frequently reported drivers for switching, highlighting the challenges associated with maintaining stable anticoagulation with VKAs in routine clinical practice [47, 48]. In addition to inadequate anticoagulation control, several studies identified treatment burden, frequent monitoring requirements, dietary restrictions, and patient or physician preference as important contributors to switching decisions [49, 50]. These findings suggest that the transition to DOAC therapy is often motivated not only by clinical considerations, but also by efforts to improve treatment convenience, patient satisfaction, and overall quality of care [51]. Nevertheless, patients switched because of unstable anticoagulation or previous adherence difficulties may remain vulnerable to suboptimal adherence behaviors even after transitioning to DOACs [52]. This pattern may partially explain why some studies continued to report moderate persistence or variable adherence despite the simplified management associated with DOAC therapy. These findings emphasize that switching anticoagulant therapy should not be considered an isolated intervention, but rather part of a broader patient-centered management strategy that includes education, follow-up, and ongoing adherence support [53].

The generally favorable adherence outcomes reported across the included studies may partially reflect several practical and treatment-related advantages associated with DOAC therapy compared with VKAs [54, 55]. Unlike VKAs, DOACs are administered using fixed-dose regimens and are associated with fewer dietary restrictions, fewer clinically significant drug interactions, and no requirement for routine INR monitoring, all of which may reduce treatment burden and improve patient convenience [56]. Adherence estimates varied substantially depending on the assessment method used. Objective measures such as the PDC, MPR, and pharmacy refill records primarily assess medication acquisition rather than actual medication intake and may therefore overestimate true adherence. In contrast, self-reported measures more directly reflect medication-taking behavior but are susceptible to recall and social desirability bias. Accordingly, adherence estimates derived from different assessment methods should be interpreted cautiously and not considered directly comparable.

Studies utilizing subjective instruments such as MMAS-8, ACTS, and PACT-Q2 generally reported lower or more variable adherence levels compared with claims-based studies, likely reflecting differences in patient perception, recall bias, and reporting behavior [37]. This variability highlights the multidimensional nature of medication adherence as described in the ABC taxonomy, in which adherence implementation and persistence may be captured differently depending on the assessment approach used Importantly, many patients included in these studies were switched because of poor INR control or unstable anticoagulation while receiving VKAs, suggesting that underlying behavioral and treatment-related adherence challenges may persist despite transitioning to a more convenient anticoagulant regimen [57]. These findings indicate that although switching to DOACs may improve treatment acceptability and simplify anticoagulation management, switching alone may not fully eliminate barriers to optimal long-term adherence [58, 59], particularly among high-risk or previously non-adherent patients [60].

Although adherence reflects the extent to which patients take medications as prescribed, persistence represents the continuation of therapy over time and therefore provides additional insight into long-term anticoagulation behavior after switching from VKAs to DOACs [61]. This distinction is consistent with the ABC taxonomy of medication adherence, which conceptualizes adherence and persistence as related but distinct constructs. In the present review, persistence outcomes were generally favorable during early follow-up periods but tended to decline gradually over time, particularly in studies with extended follow-up durations. This observation is clinically important, as discontinuation or prolonged interruption of DOAC therapy may rapidly reduce anticoagulation protection because of the relatively short half-lives of these agents compared with VKAs [62]. Several studies suggested that improved treatment convenience and reduced monitoring burden may initially support persistence after switching; however, maintaining long-term therapy appears to depend on additional factors beyond regimen simplicity alone [63–66]. These may include patient understanding of treatment importance, ongoing clinical follow-up, medication affordability, and continuity of care [67]. Accordingly, persistence should not be viewed solely as a pharmacological advantage of DOAC therapy, but rather as a multifactorial outcome influenced by behavioral, clinical, and healthcare-system-related factors that continue to affect patients even after transitioning from VKAs.

The adherence and persistence estimates observed following switching from VKAs to DOACs appear broadly consistent with those reported in the wider literature evaluating patients initiating DOAC therapy. Although previous exposure to VKA therapy may provide greater familiarity with long-term anticoagulation, the available evidence does not suggest that switching alone consistently results in superior medication-taking behavior [68]. Similarly, recent evidence suggests that the clinical consequences of switching may also vary according to patient characteristics. For example, a nationwide Korean cohort study of frail elderly Asian patients with atrial fibrillation who were clinically stable on warfarin reported that switching to DOACs was associated with higher risks of major bleeding, thromboembolic events, net clinical outcome, and all-cause mortality compared with continued warfarin therapy [69]. These findings indicate that while favorable adherence and persistence may be achieved after switching, this should not be interpreted as evidence that switching is universally associated with improved clinical outcomes. Because few primary studies directly compared patients switching from VKAs with de novo DOAC initiators, the independent effect of previous VKA experience on long-term adherence and persistence remains uncertain and warrants further investigation.

The findings of the present review provide several important clinical implications for the management of patients transitioning from VKAs to DOACs. The decline in persistence observed in several studies despite initially favorable adherence after switching suggests that the first year following transition represents a critical period for maintaining long-term anticoagulation therapy and that simplifying anticoagulation alone may be insufficient to ensure sustained treatment continuation. Patients switched because of unstable INR control or poor TTR previous adherence difficulties, or multiple comorbidities may represent high-risk subgroups requiring closer clinical monitoring and individualized adherence support after switching. Accordingly, post-switch management strategies should extend beyond medication selection and include scheduled follow-up during the first year after switching, particularly for patients with previous anticoagulation instability or other adherence-related risk factors, when persistence decline appears most likely to emerge [70]. Clinical assessment should also consider anticoagulation-specific factors such as renal function, dose appropriateness, bleeding risk, potential drug interactions, and indication-specific treatment duration when optimizing long-term DOAC management [71]. In addition, the discrepancy between objective refill-based measures and self-reported adherence assessments indicates that reliance on patient-reported adherence alone may inadequately identify patients at risk of treatment interruption [72]. Integrating objective adherence monitoring approaches (e.g., PDC or pharmacy refill records) into routine anticoagulation follow-up may facilitate earlier identification of patients at risk of treatment interruption than relying solely on patient self-report [73]. Routine DOAC-specific laboratory assays are not recommended; however, selective testing may be useful when non-adherence or altered drug exposure is suspected and should complement, rather than replace, clinical assessment. The marked variability in adherence and persistence definitions across the included studies also highlights the need for standardized outcome reporting in future anticoagulation research, particularly in studies evaluating switching strategies [74]. Future prospective studies should focus evaluate targeted adherence interventions during the early post-switch period and determine which strategies are most effective for patients at greatest risk of non-persistence following transition from VKAs to DOACs [75].

The present review has several strengths. To the best of our knowledge, this is among the first systematic reviews specifically evaluating adherence and persistence outcomes following switching from VKAs to DOACs, rather than assessing DOAC adherence in general anticoagulated populations. The inclusion of diverse study designs and healthcare settings provided a broader understanding of real-world anticoagulation practices and switching patterns. In addition, incorporating patient-reported measures and reasons for switching offered further insight into factors influencing anticoagulation behavior after transition to DOAC therapy. Nevertheless, several limitations should be acknowledged. Most included studies were observational in nature and susceptible to confounding and selection bias. Considerable heterogeneity in adherence definitions, persistence methodologies, follow-up duration, and clinical outcome reporting limited direct comparison across studies and precluded quantitative meta-analysis. Additionally, the literature search was limited to PubMed and Scopus and did not include other databases or grey literature sources. Therefore, some relevant studies indexed exclusively in databases such as Embase, Web of Science, CINAHL, the Cochrane Library, or regional databases may not have been identified. Many studies also lacked pre- and post-switch adherence comparisons within the same patient population, limiting conclusions regarding whether switching directly improved adherence or persistence. Furthermore, many adherence estimates were derived from refill- or claims-based measures, which assess medication acquisition rather than actual medication intake. Consequently, these measures may overestimate true adherence, and the reported estimates should be interpreted within the context of the assessment methods used. Variability in healthcare systems, reimbursement policies, affordability, insurance coverage, drug accessibility, and patient populations across countries may have influenced both medication refill behavior and long-term persistence, thereby limiting the generalizability of the findings across different healthcare settings. Additionally, AF and VTE populations were synthesized together despite important differences in recommended anticoagulation duration. Whereas AF generally requires lifelong anticoagulation, VTE is frequently managed with time-limited therapy depending on the provoking factors and recurrence risk. Because most primary studies did not report outcomes separately by indication, indication-specific synthesis was not feasible. Consequently, persistence outcomes should be interpreted with caution, as differences in treatment duration may have contributed to the observed heterogeneity. Finally, only English-language studies were included, and publication bias could not be formally assessed because of the methodological heterogeneity and limited number of studies available for each outcome.

## Conclusion

The available evidence indicates that patients who switched from VKAs to DOACs generally demonstrated moderate-to-high adherence and persistence during the post-switch period, particularly during the early period following transition. However, substantial variability was observed across studies, largely driven by differences in adherence assessment methods, persistence definitions, follow-up duration, and patient populations. Current evidence describes post-switch medication-taking patterns rather than demonstrating that switching itself improves adherence or persistence. Patients switched because of poor INR control or unstable anticoagulation may remain vulnerable to long-term adherence and persistence challenges despite transitioning to a simplified anticoagulation regimen. These findings highlight the importance of continued follow-up and adherence-support strategies after switching rather than considering the transition itself as sufficient to optimize long-term anticoagulation behavior. Future well-designed prospective studies using standardized adherence and persistence definitions, longer follow-up, and direct pre- and post-switch comparisons are needed to better understand the impact of switching from VKAs to DOACs and to support the development of targeted interventions for improving long-term anticoagulation outcomes.

## Supplementary Information

Supplementary Material 1.

Supplementary Material 2.

## Data Availability

All data presented in the current work are available in the text and supplementary materials.

## Abbreviations

ACTS: Anti-Clot Treatment Scale
AF: Atrial fibrillation
AXIS: Appraisal Tool for Cross-Sectional Studies
DOAC: Direct oral anticoagulant
DVT: Deep vein thrombosis
EHR: Electronic health record
INR: International normalized ratio
LMWH: Low-molecular-weight heparin
MMAS-8: 8-item morisky medication adherence scale
MPR: Medication possession ratio
NOS: Newcastle–Ottawa Scale
PACT-Q2: Perception of Anticoagulant Treatment Questionnaire
PDC: Proportion of days covered
PE: Pulmonary embolism
PICOS: Population, intervention, comparator, outcomes, and study design
PRISMA: Preferred Reporting Items for Systematic Reviews and Meta-Analyses
RCT: Randomized controlled trial
RoB 2: Risk of Bias Tool version 2
TTR: Time in therapeutic range
VKA: Vitamin K antagonist
VTE: Venous thromboembolism
