# Supplementary material for: Medication adherence and treatment persistence following switching from vitamin K antagonists to direct oral anticoagulants: a systematic review
Source: BMC Cardiovasc Disord. 2026 Aug 1;26:991. doi: 10.1186/s12872-026-06398-0 (PMC13718113; doi:10.1186/s12872-026-06398-0)
Supplement: Supplementary file 2 [file 12872_2026_6398_MOESM2_ESM.docx]

**Supplementary File 2**

**Table S1:** Search strategies

|  | **Search Terms** |
| --- | --- |
| **PubMed** | |
| #1 | ("Warfarin"[Mesh] OR warfarin[tiab] OR coumadin[tiab] OR "vitamin K antagonist*"[tiab] OR VKA[tiab] OR acenocoumarol[tiab] OR phenprocoumon[tiab] OR fluindione[tiab]) |
| #2 | ("Anticoagulants"[Mesh] OR "Factor Xa Inhibitors"[Mesh] OR "Antithrombins"[Mesh] OR "Dabigatran"[Mesh] OR "Rivaroxaban"[Mesh] OR "Apixaban"[Mesh] OR "Edoxaban"[Supplementary Concept] OR apixaban[tiab] OR rivaroxaban[tiab] OR dabigatran[tiab] OR edoxaban[tiab] OR betrixaban[tiab] OR "direct oral anticoagulant*"[tiab] OR "novel oral anticoagulant*"[tiab] OR "non vitamin K oral anticoagulant*"[tiab] OR "new oral anticoagulant*"[tiab] OR DOAC[tiab] OR NOAC[tiab] OR TSOAC[tiab] OR "factor Xa inhibitor*"[tiab] OR "direct thrombin inhibitor*"[tiab] OR xarelto[tiab] OR eliquis[tiab] OR pradaxa[tiab] OR savaysa[tiab]) |
| #3 | ("Medication Adherence"[Mesh] OR "Patient Compliance"[Mesh] OR adheren*[tiab] OR complian*[tiab] OR persist*[tiab] OR discontinu*[tiab] OR continuation[tiab] OR interruption[tiab] OR cessation[tiab] OR concordance[tiab] OR refill*[tiab] OR "medication possession ratio"[tiab] OR MPR[tiab] OR "proportion of days covered"[tiab] OR PDC[tiab] OR "drug utilization"[tiab] OR "medication use"[tiab] OR "drug survival"[tiab] OR "long-term adherence"[tiab] OR "adherence trajectory"[tiab]) |
| #4 | (switch*[tiab] OR transition*[tiab] OR convert*[tiab] OR chang*[tiab] OR crossover[tiab] OR "change of therapy"[tiab] OR "treatment switch"[tiab]) |
| #5 | #1 AND #2 AND #3 AND #4 |
| **Scopus** | |
| #1 | (TITLE-ABS-KEY(warfarin OR coumadin OR "vitamin K antagonist*" OR VKA OR acenocoumarol OR phenprocoumon OR fluindione) |
| #2 | TITLE-ABS-KEY(anticoagulants OR "factor Xa inhibitor*" OR antithrombin* OR dabigatran OR rivaroxaban OR apixaban OR edoxaban OR betrixaban OR "direct oral anticoagulant*" OR "novel oral anticoagulant*" OR "non vitamin K oral anticoagulant*" OR "new oral anticoagulant*" OR DOAC OR NOAC OR TSOAC OR "direct thrombin inhibitor*" OR xarelto OR eliquis OR pradaxa OR savaysa) |
| #3 | TITLE-ABS-KEY(adheren* OR complian* OR persist* OR discontinu* OR continuation OR interruption OR cessation OR concordance OR refill* OR "medication possession ratio" OR MPR OR "proportion of days covered" OR PDC OR "drug utilization" OR "medication use" OR "drug survival" OR "long-term adherence" OR "adherence trajectory") |
| #4 | TITLE-ABS-KEY(switch* OR transition* OR convert* OR chang* OR crossover OR "change of therapy" OR "treatment switch") |
| #5 | #1 AND #2 AND #3 AND #4 |

**Table S2:** Clinical and treatment-related factors evaluated for their association with adherence and persistence following switching from vitamin k antagonists to direct oral anticoagulants

| **Study** | **Population** | **Outcome** | **Factors Evaluated** | **Main Findings** |
| --- | --- | --- | --- | --- |
| Elling et al., 2024 [34] | AF+VTE switched VKA→DOAC | Adherence & Persistence | Previous INR control (TTR/TUR), INR variability, age, sex, VKA duration, frailty, DOAC type, switching, antiplatelet therapy | Previous INR control was not associated with subsequent adherence or persistence. |
| Toorop et al., 2022 [33] | NVAF | Persistence | Pre-switch TTR, age, sex, anticoagulation clinic, CHA₂DS₂-VASc, HAS-BLED, comorbidities | Low pre-switch TTR predicted poorer persistence. |
| Inoue et al., 2016 [45] | NVAF | Adherence | Age, renal function, dabigatran dose, switch from warfarin, concomitant medications | No formal predictor analysis performed. |
| Yang et al., 2020 [47] | Adults with congenital heart disease | Adherence | MMAS-8, refill adherence, prior VKA use, quality of life | No predictor analysis performed. |
| Hanon et al., 2016 [36] | NVAF switched VKA→rivaroxaban | Persistence, satisfaction, adherence | Age, ACTS, quality of life, physician satisfaction | No multivariable predictor analysis performed. |
| Bartoli-Abdou et al., 2021 [40] | AF/VTE switched for low TTR | Long-term adherence | Medication beliefs, illness beliefs, treatment burden, hypertension, deprivation, bleeding history, comorbidity burden | Medication beliefs and treatment burden significantly influenced adherence. |
| Keita et al., 2017 [38] | VTE | Adherence, QoL | Anticoagulant type, treatment duration, physician–patient relationship, patient involvement | No statistical predictor analysis; physician relationship emphasized. |
| Benzimra et al., 2018 [37] | AF | Adherence, satisfaction | Treatment type, convenience, treatment satisfaction | No independent predictors identified. |
| Toorop et al., 2020 [32] | Switched VKA→DOAC | Adherence & Persistence | Age, sex, education, consultation frequency, VKA duration, INR monitoring, bleeding history, dosing frequency, specialist follow-up | Younger age, twice-daily dosing, previous bleeding and limited specialist follow-up associated with poorer adherence. |
| Serrao et al., 2020 [39] | AF/recurrent VTE | Adherence | Treatment satisfaction, QoL, missed doses, DOAC plasma levels | No formal predictor analysis performed. |
| Lee et al., 2021 [46] | NVAF | Treatment continuation | Age, sex, comorbidity, treatment type, convenience, satisfaction | No independent predictors identified. |
| Pundi et al., 2021 [41] | Veterans with AF | Objective adherence (PDC) | Pre-switch TTR, age, race, sex, renal function, comorbidity, medication burden | Low pre-switch TTR independently associated with poorer adherence. |
| Pham Nguyen et al., 2020 [42] | AF after hospitalization | Objective adherence | Hospitalization, bleeding admission, switching, age, race, education, income, frailty | Bleeding-related hospitalization associated with reduced adherence. |
| Mews et al., 2021 [43] | AF/VTE/other indications | Persistence & refill adherence | Eligibility assessment, renal function, body weight, drug interactions, indication, insurance, cost | No formal predictor analysis; cost identified as major practical barrier. |
| Douros et al., 2017 [44] | NVAF | Persistence | Age, thromboembolic risk, cardiovascular disease, bleeding history, kidney/liver disease, cancer, anticoagulant type | Older age and higher CHA₂DS₂-VASc associated with greater persistence. |
| Inoue et al., 2016 [45] | Japanese NVAF | Early adherence | Age, renal function, dose, prior warfarin, comorbidities, concomitant medications | Clinical characteristics reported descriptively only; no determinant analysis. |

**Abbreviations:** AF = atrial fibrillation; ACTS = Anti-Clot Treatment Scale; CHA₂DS₂-VASc = Congestive heart failure, Hypertension, Age ≥75 years, Diabetes mellitus, Stroke/transient ischemic attack/thromboembolism, Vascular disease, Age 65–74 years, Sex category (female); DOAC = direct oral anticoagulant; HAS-BLED = Hypertension, Abnormal renal/liver function, Stroke, Bleeding history or predisposition, Labile international normalized ratio, Elderly, Drugs/alcohol concomitantly; INR = international normalized ratio; MMAS-8 = 8-item Morisky Medication Adherence Scale; NVAF = non-valvular atrial fibrillation; PDC = proportion of days covered; QoL = quality of life; TTR = time in therapeutic range; TUR = time under therapeutic range; VKA = vitamin K antagonist; VTE = venous thromboembolism.

**Table S3:** Methodological quality assessment of cross-sectional studies using the AXIS tool

| **Study** | **Clear Objectives** | **Appropriate Sampling** | **Valid Measurement** | **Non-response Bias Addressed** | **Appropriate Analysis** | **Main Concerns** | **Overall Interpretation** |
| --- | --- | --- | --- | --- | --- | --- | --- |
| Toorop et al., 2020 [27] | Yes | Moderate | Yes | No | Yes | Self-report bias, selection bias | Moderate quality |
| Lee et al., 2021 [41] | Yes | Moderate | Yes | No | Yes | Survey-based, non-response bias | Moderate quality |
| Benzimra et al., 2018 [32] | Yes | Moderate | Yes | No | Yes | Small sample, self-reported data | Moderate quality |
| Keita et al., 2017 [33] | Yes | Low | Yes | No | Yes | Very small sample, selection bias | Moderate quality |
